# The Impact of SNP-Induced Amino Acid Substitutions L19P and G66R in the dRP-Lyase Domain of Human DNA Polymerase β on Enzyme Activities

**DOI:** 10.3390/ijms25084182

**Published:** 2024-04-10

**Authors:** Olga A. Kladova, Timofey E. Tyugashev, Denis V. Yakimov, Elena S. Mikushina, Daria S. Novopashina, Nikita A. Kuznetsov, Aleksandra A. Kuznetsova

**Affiliations:** 1 Institute of Chemical Biology and Fundamental Medicine, Siberian Branch of Russian Academy of Sciences, Novosibirsk 630090, Russianikita.kuznetsov@niboch.nsc.ru (N.A.K.); 2 Department of Natural Sciences, Novosibirsk State University, Novosibirsk 630090, Russia

**Keywords:** DNA repair, DNA polymerase beta, single-nucleotide polymorphism, enzymatic activity

## Abstract

Base excision repair (BER), which involves the sequential activity of DNA glycosylases, apurinic/apyrimidinic endonucleases, DNA polymerases, and DNA ligases, is one of the enzymatic systems that preserve the integrity of the genome. Normal BER is effective, but due to single-nucleotide polymorphisms (SNPs), the enzymes themselves—whose main function is to identify and eliminate damaged bases—can undergo amino acid changes. One of the enzymes in BER is DNA polymerase β (Polβ), whose function is to fill gaps in DNA. SNPs can significantly affect the catalytic activity of an enzyme by causing an amino acid substitution. In this work, pre-steady-state kinetic analyses and molecular dynamics simulations were used to examine the activity of naturally occurring variants of Polβ that have the substitutions L19P and G66R in the dRP-lyase domain. Despite the substantial distance between the dRP-lyase domain and the nucleotidyltransferase active site, it was found that the capacity to form a complex with DNA and with an incoming dNTP is significantly altered by these substitutions. Therefore, the lower activity of the tested polymorphic variants may be associated with a greater number of unrepaired DNA lesions.

## 1. Introduction

DNA molecules are essential to life because they contain information on the structure of an organism’s proteins. Alterations in a normal sequence of nucleotides in DNA can result from endogenous and external factors [1,2,3,4]. Indeed, numerous processes, including oxidation, alkylation, deamination of nitrogenous bases, apurinic/apyrimidinic site formation, and DNA strand breakage, can result in damage to the DNA molecule [5,6,7,8].

Base excision repair (BER), which involves the sequential action of DNA glycosylases, apurinic/apyrimidinic endonucleases, DNA polymerases, and DNA ligases, is one of several enzymatic systems that maintain genome integrity [9,10,11,12]. DNA polymerase β (Polβ), one of the BER enzymes, fills DNA gaps with complementary dNMPs and eliminates the 2′-deoxyribose phosphate residue at the 5′- end of a DNA strand break [13,14,15,16,17]. In addition to these processes, Polβ is engaged in non-homologous end joining and telomere repair, which both contribute to genome integrity [18,19,20,21,22]. Although BER functions effectively in the normal state, sequence changes in the *POLΒ* gene itself owing to natural single-nucleotide polymorphisms (SNPs) may yield an impaired enzyme [23].

In the genes that code for DNA repair enzymes, SNPs are common [24,25,26]. Many naturally occurring polymorphic forms of Polβ are known to be less active than the wild-type (WT) enzyme [27,28,29,30,31,32]. Additionally, it has been determined that up to 30% of examined human malignant tumors have Polβ variants [33]. Several databases contain information about different Polβ variants in the dRP-lyase domain that have been found in patients with various types of cancer (Table 1). Many of these mutations are class switching, indicating a possible role of certain amino acid substitutions in cancer predisposition.

Switching a class of an amino acid residue can cause a change in its function, thereby affecting its activity. Previously, Polβ polymorphisms that cause a change in the class of an amino acid residue were analyzed using online software [34]. In the present study, we considered those substitutions that were found only in the dRP-lyase domain. It was revealed that 50 variants are located in the dRP-lyase domain, which is formed by amino acid residues (aa) 1–87. They were ranked according to the degree of possible influence on the functioning of Polβ. Of these, five polymorphic variants, namely substitutions L19P, L22P, K35E, A42T, and G66R, were predicted to pose a significant risk of impairing Polβ function. There are some literature data concerning how some of these polymorphic variations affect Polβ activity. For instance, it has been demonstrated that the Polβ L22P enzyme lacks dRP-lyase activity while retaining transferase activity [35]. Furthermore, the L22P substitution decreases the efficiency of the enzyme binding to DNA. Notably, though the catalytic amino acid residue necessary for the dRP-lyase reaction is Lys-72, the replacement of Leu-22 with Pro eliminates this activity. Another mutation leading to the replacement of Leu-22 with Phe has been found in patients with bile duct adenocarcinoma (Table 1). When we compared the list of mutant variants with the available information about oncological SNPs in Polβ (Table 1, three databases) there was no overlap between the data, indicating that, at this moment, databases do not fully reflect information about known polymorphic substitutions and tumorigenesis. Therefore, this work was aimed at studying new SNPs that, according to theoretical predictions, can contribute to changes in the function of Polβ.

This research was designed to determine how two SNP-associated amino acid substitutions (L19P and G66R, which had not been described previously) would affect the enzymatic activity of Polβ. Despite there being little information on the occurrence of these sequence variants in cancer in the literature or databases, it was demonstrated [34] that both variants pose an elevated risk of impaired Polβ function.

**Figure 1:**
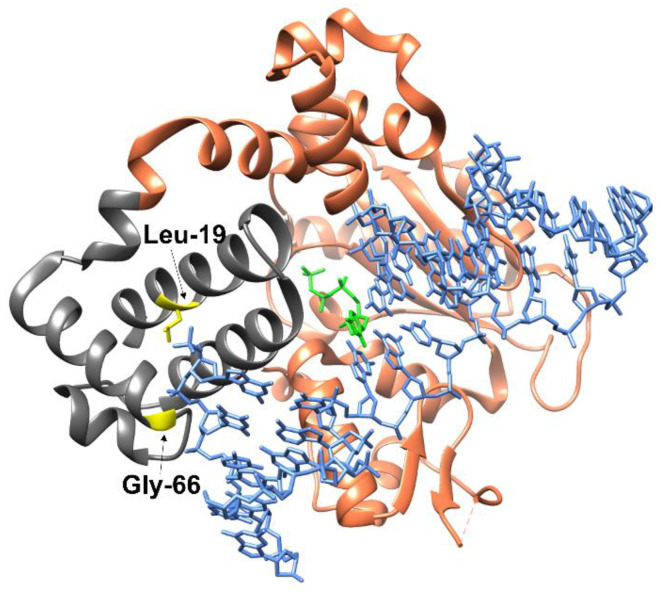
Structure of Polβ in the complex with DNA containing a 1 nt gap and a modified dCTP analog. Polβ is highlighted in orange, the dRP-lyase (aa 1–87) domain is gray, Leu-19 and Gly-66 are yellow, the modified dCTP analog is green, and DNA is blue. Protein Data Bank (PDB) ID: 5UGP.

The substitutions in question are located in the dRP-lyase domain of Polβ, with both amino acid residues near the 3′ end of the DNA strand (Figure 1). Residue Leu-19 is located in the α1 helix (aa 13–28), and upon binding to DNA, it appears near the 5′ end of the damaged DNA strand containing the gap. Amino acid residue Gly-66 is located in the α4-helix and is a part of one of two DNA-binding helix–hairpin–helix motifs (aa 55–79), which provide nonspecific interactions with the sugar–phosphate backbone of DNA that are necessary to stabilize the pronounced DNA bend (~90°) upon formation of the complex with Polβ [36].

## 2. Results and Discussion

### 2.1. Determining the Effect of Amino Acid Substitutions on the Thermal Stability and Secondary Structure of Polβ

**Figure 2:**
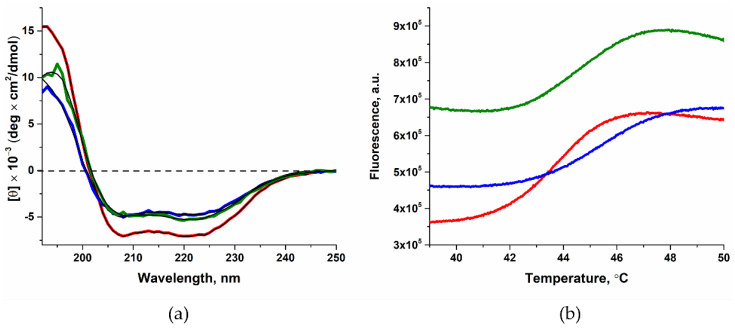
Checking the effect of substitutions L19P (blue) and G66R (green) on Polβ secondary structure and thermal stability. Data on WT Polβ are shown in red. (**a**) CD spectra, (**b**) melting curves. The full-length enzymes were used. The fitting line is indicated by a solid black line.

Full-length variants of Polβ were analyzed by circular dichroism (CD) spectroscopy to evaluate the content of α-helices in the protein. The obtained CD spectra (Figure 2a) differed little from the data obtained from the WT enzyme. The calculated contents of α-helices, taking into account measurement error, were also similar between the enzymes (Table 2). Additionally, thermal stability of the enzyme variants was analyzed by the thermal shift method using the fluorescent dye ProteOrange (Figure 2b). The resulting melting curves were analyzed via Equation (1). The melting temperature obtained for the enzyme variants was almost the same as that for the WT enzyme (Table 2). Taken together, these data indicated a negligible effect of the L19P and G66R amino acid substitutions on the total content of secondary elements in the protein structure and on the thermal stability of the protein globule.

Indeed, although the G66R substitution (located in a loop region of a helix-3-turn-helix-4 region) does not influence the overall domain structure, substitution L19P (located in the helix-1 region) affects one of the residues forming the hydrophobic core of the dRP-lyase domain [37]. Loss of the hydrophobic contacts previously provided by a helix-stabilizing leucine sidechain, together with proline-induced destabilization, may result in unfolding or kinking of the normal domain structure [38,39]. Notionally similar, but positioned deeper in the α-helix, tumor-associated substitution L22P is reported to result in an unfolded dRP-lyase domain, albeit one restored by protein–DNA complex formation [40]. Based on these data, it could be hypothesized that these mutations can introduce local changes that cannot be detected by the above experimental methods. Therefore, a molecular dynamics (MD) simulation was performed to assess the local conformational alterations induced by these substitutions.

**Table 2:** Contents of α-helices and calculated melting points of the enzymes.

	WT Polβ	Polβ L19P	Polβ G66R
Content of α-helices, %	79 ± 16 *	55 ± 11	66 ± 13
T_m_, °C	44.9 ± 0.2 **	45.3 ± 0.1	44.6 ± 0.1

* Data from ref. [41]; ** data from ref. [42].

**Figure 3:**
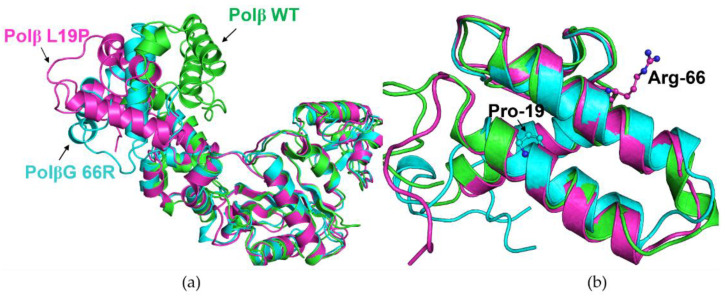
Overlay of representative snapshots of the simulations of the apoenzyme (**a**) and dRP-lyase domain (**b**). The WT protein is highlighted in green, the L19P variant in cyan, and the G66R variant in magenta.

Simulations of full apoenzyme Polβ suggested that both the WT and the polymerase variants maintain their extended conformations, with N-terminal dRP-lyase and finger domains motile on their flexible hinges relative to palm and finger domains (Figure 3a). In simulations of the dRP-lyase domain alone, the L19P substitution yielded partial destabilization of the helix-1 region, whereas the G66R substitution with the sidechain oriented to the solution did not affect overall domain structure (Figure 3b).

### 2.2. Determination of the Dissociation Constant for the Enzymes and DNA

**Figure 4:**
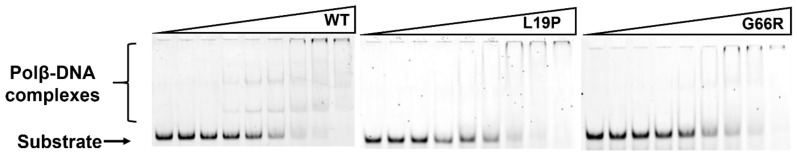
DNA-binding efficiency of WT Polβ and of polymorphic variants L19P and G66R toward substrate Gap_C. The studied Polβ variants showed less effective complex formation with the DNA substrate. For the L19P variant, a clear-cut band related to the complex of the enzyme with DNA was undetectable.

To test whether the decrease in polymerase activity is associated with an effect of the amino acid substitutions on the efficiency of binding to DNA, an electrophoretic mobility shift assay (EMSA) was carried out. DNA substrates Gap_A, Gap_T, Gap_G, and Gap_C contained a 1 nt gap with a different nucleotide placed in the opposite strand and were FAM-labeled on the elongated strand. The different complementary DNA strands, which allowed placing different nucleotides opposite the 1 nt gap, were used to check the influence of various bases on the efficiency of binary enzyme–DNA complex formation (Figure 4).

**Figure 5:**
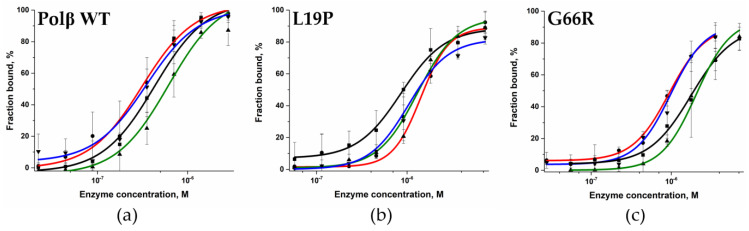
Complex formation efficiency of WT Polβ (**a**) and of polymorphic variants L19P (**b**) and G66R (**c**) toward DNA substrates containing different nucleotides opposite the 1 nt gap [black: Gap_A (■), red: Gap_T (●), green: Gap_G (▲), and blue: Gap_C (▼)]. WT Polβ concentration: 2.83 μM to 22 nM, L19P concentration: 57 nM to 7.3 μM, G66R concentration: 27.7 nM to 3.55 μM (in experiments with Gap_T and Gap_C) and 55.5 nM to 7.1 μM (in experiments with Gap_A and Gap_G). The DNA concentration was 50 nM.

Analysis of the EMSA gels using GelPro4 software (version 4.0) revealed the proportion of each DNA substrate bound to the enzyme at different enzyme concentrations. The dependence of the proportion of the bound DNA substrate on the enzyme concentration had a hyperbolic shape (Figure 5) and made it possible to calculate dissociation constants of polymorphic variants toward all types of DNA substrates using Equation (2) (Table 3).

**Figure 6:**
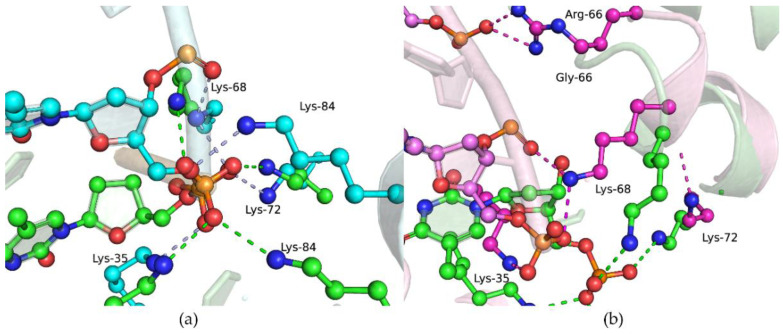
Overlays of snapshots for the dRP-lyase domain in an open binary protein–DNA complex. WT (green) and L19P (cyan) overlay (**a**). WT (green) and G66R (magenta) overlay (**b**). Salt bridges between amino acid sidechains and the sugar–phosphate backbone are shown as dashed lines.

Analysis of these data (Table 3) revealed that the L19P polymorphic variant binds to DNA on average 3–4 times less effectively than the WT enzyme does. The findings supported the MD simulations, which indicated that replacing Leu-19 with the more compact Pro residue leads to partial unfolding of the α1-helix and disruption of the interaction between other amino acid residues (Figure 6a). Indeed, despite Leu-19 being located away from the DNA-binding site, its replacement with Pro-19 leads to a change in the position of the α1-helix, and subsequent rearrangement in α2- and α3-helices. These rearrangements lead to local replacement of several Lys residues (Lys-35, Lys-68, Lys-72, Lys-84), resulting in their relocation relative to the 5′-end of the DNA primer while maintaining hydrogen bonds with the oxygen atoms of the phosphate groups. Nevertheless, these distortions of the dRP-lyase domains’ α-helical structure most likely alter the 5′-phosphate–binding site.

For the G66R polymorphic variant, weaker affinity for DNA was shown too (Table 3), which may be explained by the appearance of bulky charged residue Arg-66 at the position of Gly-66. The latter provides nonspecific interactions with the sugar–phosphate DNA backbone that are necessary for stabilization of pronounced bending of DNA (~90°) during the formation of a complex with Polβ [36]. In simulations of the G66R variant, the Arg-66 sidechain formed salt bridges with a phosphate group of a downstream DNA nucleotide, thereby causing displacement of the 5′-phosphate termini from their original binding site Lys35-Lys72-Lys84.

Taken together, these data suggested that local disruption of the contacts of dRP-lyase domain amino acid residues led to less stable and less efficient binding of the studied Polβ variants to DNA. Thus, both natural polymorphic variants of Polβ have weaker DNA-binding affinity compared to the WT enzyme.

### 2.3. Determination of the dRP-Lyase Activity of the Polβ Variants

**Figure 7:**
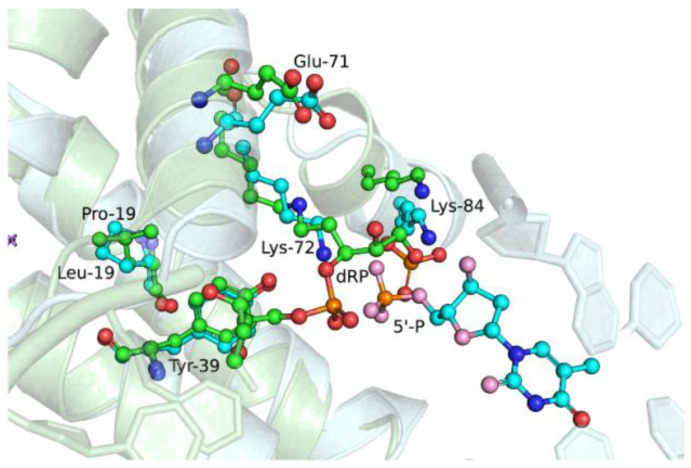
Overlay of structures for simulated open binary complex of L19P variant with DNA (cyan) and trapped complex of WT Polβ with dRP-containing DNA (green, PDB ID: 7RBE).

**Figure 8:**
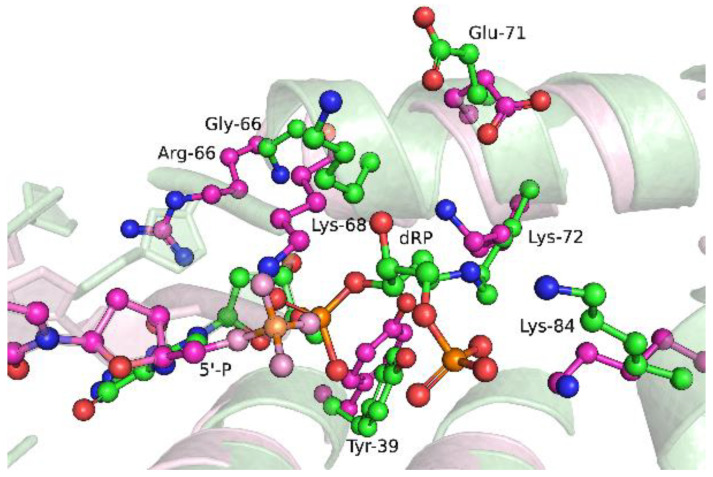
Overlay of structures for simulated open binary complex of G66R variant with DNA (magenta) and trapped complex of WT Polβ with dRP-containing DNA (green, PDB ID: 7RBE).

Polβ has dRP-lyase activity, which has been intensively studied in many works [43,44,45,46,47,48,49,50,51]. The sequence of chemical transformations in the presence of a dRP residue in DNA has remained not entirely clear. In ref. [52], it was shown that the Schiff base between dRP residue and catalytic Lys-72 is formed before the transferase reaction occurs. In order to evaluate the possible influence of L19P and G66R variants on dRP-lyase reaction efficiency, we performed a comparison of the crystal structure of a trapped Polβ-DNA complex (PDB ID: 7RBE) reported in [52] and structures containing substitutions of amino acid residues obtained from molecular dynamics modeling (Figure 7 and Figure 8).

When the structures of the open binary complex of the L19P variant and trapped WT Polβ-DNA complex (Figure 7) were superimposed, a slight displacement of Lys-72 from its original position occurred due to the influence of Pro-19 on the structure of the α1-helix. Moreover, previously, it was shown that Tyr-39 and Glu-71 play an important role in Schiff-base formation and β-elimination reaction [52]. Tyr-39 may stabilize the deprotonated form of Lys-72 through a hydrogen bond, whereas Glu-71 could catalyze the β-elimination reaction through a water-assisted C2′ proton abstraction. An overlay of two structures revealed the very close position of the Tyr-39 residue in these structures, but relocation of Glu-71 in the L19P variant.

Comparison of the structure of the binary DNA–G66R variant complex and the crystal structure of the trapped DNA-Polβ complex revealed the rotation of the side radical of Glu-71 while the close position of atoms in the backbone of the protein chain was maintained (Figure 8). It is interesting to note that the Tyr-39 and Lys-72 residues synchronously change position, but do not lose connection with each other. Therefore, generally, the spatial organization of the dRP-lyase active site of the G66R variant was similar to that of the WT Polβ, which may indicate minor differences in the dRP-lyase activity of the G66R variant.

**Figure 9:**
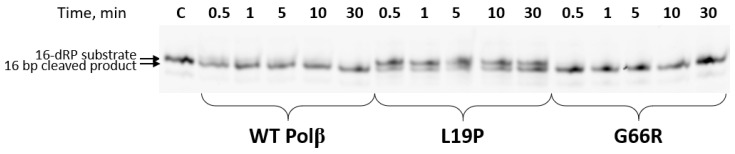
Comparison of the dRP-lyase activity of polymorphic Polβ variants. DNA concentration was 1 μM, enzyme concentration was 2.5 μM. The reaction was conducted at 20 °C. C denotes 3ʹ-FAM-labeled 16 bp dRP DNA substrate.

To check assumptions made based on the crystal structure analysis, the dRP-lyase activity of Polβ variants was tested. The dRP-lyase substrate was generated using 3′-FAM-labeled 36 bp U-containing DNA treated with Udg and APE1 enzymes. The resulting 16 bp dRP-substrate was mixed with WT Polβ, L19P, or G66R variants. The reaction was stopped at various time points and reaction mixture was loaded in 20% TBE-urea PAAG (Figure 9).

The obtained data indicate that the G66R variant has dRP-lyase activity comparable to that of the WT Polβ. It can be concluded that the introduction of the bulk Arg-66 residue does not lead to the redistribution of local contacts necessary for the dRP-lyase reaction. At the same time, another polymorphic variant, L19P, proved to have reduced dRP-lyase activity compared to the WT enzyme. This confirms the assumption that Pro-19 influences the local organization of the dRP-binding site.

### 2.4. An Assay of Primer Extension Efficiency of the Polβ Variants

**Figure 10:**
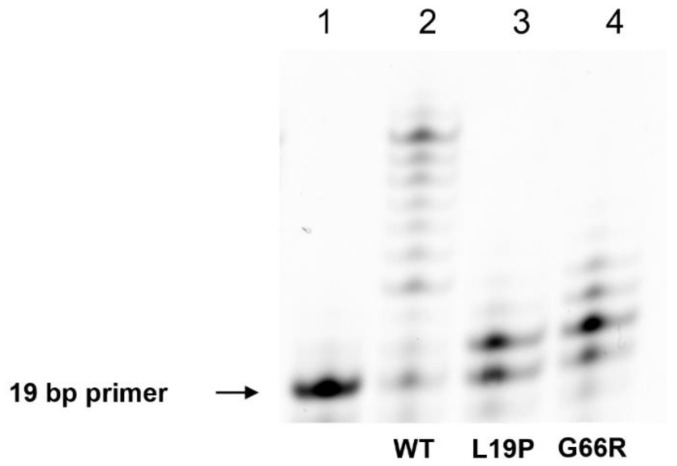
A comparison of the effectiveness of strand replacement synthesis by polymorphic Polβ variants. The 19-Nucleotide FAM-labeled DNA primer Gap_T is in lane 1, primer extension by WT Polβ is presented in lane 2, primer extension by Polβ L19P is in lane 3, and primer extension by Polβ G66R is in lane 4. The WT enzyme produced products up to 30 nt in length within 1 min of the reaction by elongating the DNA primer by up to 11 nt. Variants L19P and G66R proved to be less active polymerases compared to the WT enzyme. The L19P variant extended the primer by 3 nt. The G66R variant elongated the primer DNA by up to 4 nt. The dNTP mix concentration was 10 μM, the enzymes’ and DNA concentrations were 0.5 μM, and the temperature was 37 °C.

To analyze the influence of the studied amino acid substitutions on the polymerase activity, 6-FAM-labeled DNA substrate Gap_T was used (which mimics a single-nucleotide gap with thymidine located opposite to the gap). DNA polymerase β is most active when filling small gaps in DNA (1–2 nucleotides), but the enzyme is also capable of DNA synthesis with strand displacement. In the assay of the ability of the Polβ variants to perform strand replacement synthesis, it was demonstrated that these enzymes have reduced polymerase activity (Figure 10). Indeed, within 1 min of the reaction, the WT enzyme catalyzed the incorporation of up to 11 nucleotides into the DNA substrate containing a thymidine residue opposite the gap. By contrast, polymorphic variants L19P and G66R catalyzed incorporation of up to 3 and 4 nt, respectively. This finding means there was a negative effect of the tested substitutions on processes of strand elongation in the long-patch BER pathway.

### 2.5. Determination of the Gap-Filling Efficiency of the Polβ Variants

**Figure 11:**
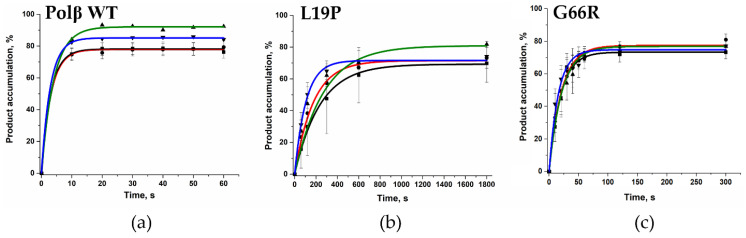
The dependence of reaction product accumulation on time for WT Polβ (**a**) [24] and for polymorphic variants Polβ L19P (**b**) and Polβ G66R (**c**). DNA substrates containing various nucleotides in the gap are highlighted with colors. Black: Gap_A (■), red: Gap_T (●), green: Gap_G (▲), and blue: Gap_C (▼). In the reaction mixture in this assay, DNA concentration was 0.5 μM, enzyme concentration was 0.5 μM, and dNTP concentration was 5 μM.

To estimate the influence of the tested amino acid substitutions on the ability of the polymorphic variants to fill single-nucleotide gaps, experiments on primer extension and strand displacement DNA synthesis were conducted next. To analyze the effects of the substitutions on the primary enzymatic function of Polβ, we performed a gap-filling assay in the presence of only complementary dNTP (Figure 11). The set of DNA substrates was the same as in the DNA-binding assay and contained various nucleotides opposite the 1 nt gap.

Kinetic traces of the product accumulation (Figure 11) were fitted to exponential Equation (3) in order to calculate characteristic observed rate constant *k*_obs_. It was revealed that the 1 nt gap filling by both polymorphic variants was more efficient when a cytidine was placed directly opposite the gap (Table 4). Of note, the WT enzyme is also known to have higher efficiency in the case of substrate Gap_C [41]. A comparison of dNTP incorporation by the polymorphic variants suggested that the G66R substitution causes at least a fivefold decrease in *k*_obs_. In contrast, the effect of L19P was much stronger and led to more than a 30-fold reduction in the enzyme activity when compared with the WT. So much lower enzymatic activity in the SNP variants cannot be explained only by changes in the enzyme’s capacity for DNA substrate binding (Table 3). Therefore, it was hypothesized that the binding of dNTP and the rate of the catalytic reaction can also influence the total enzymatic efficacy.

### 2.6. Effects of the Amino Acid Residue Substitutions on the Binding of dNTPs and Catalysis

To identify the reason for the polymerase activity decrease induced by substitutions L19P and G66R, the observed dissociation constant of the enzyme–DNA complex toward dATP and polymerization rate constant *k*_pol_ were determined. To assess the enzymes’ ability to bind the complementary 5′-deoxynucleotide triphosphate residue and the rate of the catalytic reaction, the stopped-flow method was chosen because it can be used to monitor a quick enzymatic reaction by recording the conformational changes of biopolymers that can occur during enzyme–substrate interactions in a millisecond or second time range.

It has been shown previously that a 2-aminopurine (2-aPu) fluorescent residue is a sensitive label that could help with the detection of both binding of dNTP and its incorporation into a DNA substrate [53,54,55,56]. Moreover, stopped-flow analysis of Polβ interaction with DNA and dNTPs has been well characterized by means of 2-aPu fluorescence intensity changes [41,42,57,58]. It has been stated that two-stage changes in fluorescence intensity of a 2-aPu residue correspond to (i) the stage of formation of a ternary closed complex of the enzyme, DNA, and dNTP (an increase phase) and to (ii) the chemical stage of transfer of the dNMP residue to the 3′ end of a primer and formation of the reaction product (a phase of a decrease in fluorescence intensity) [57,58,59,60].

**Figure 12:**
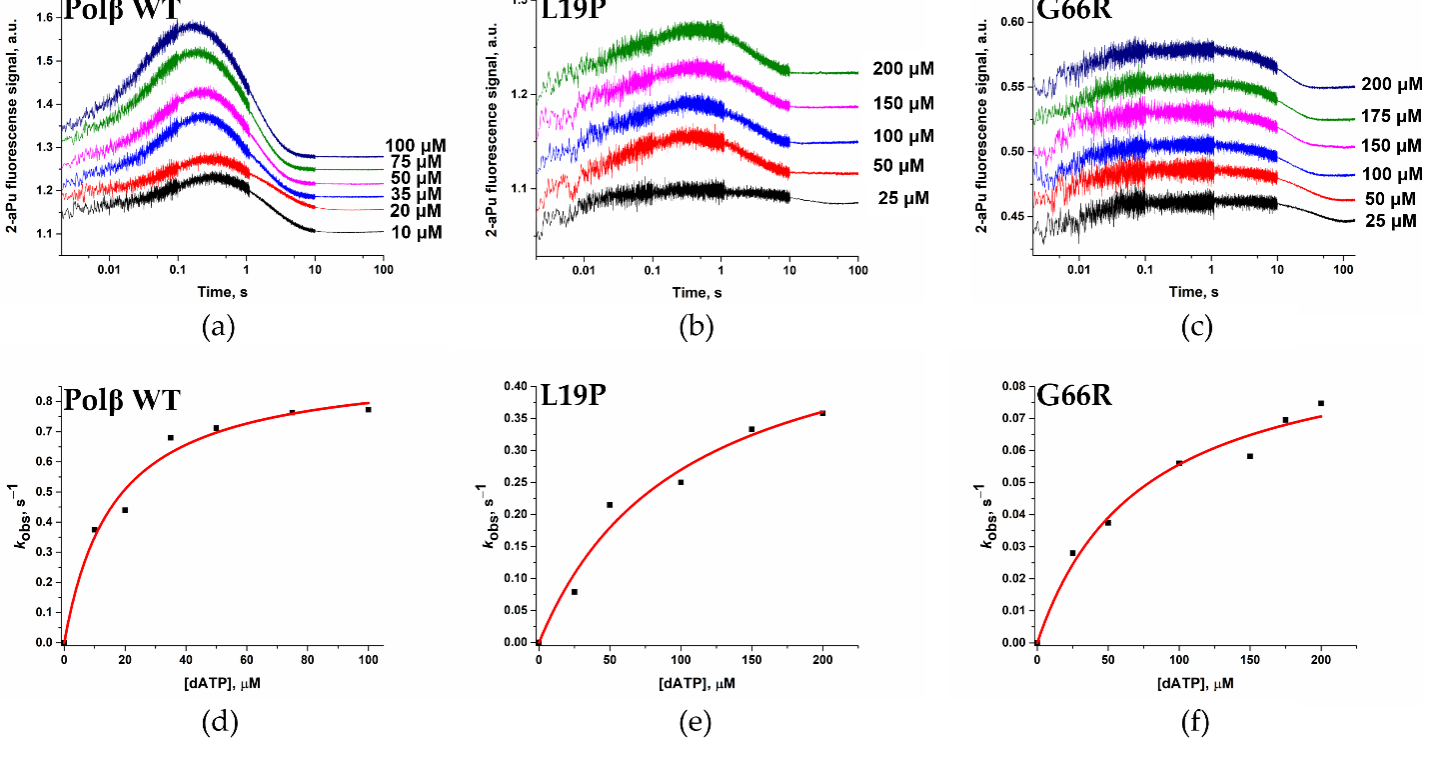
Changes in fluorescence intensity of a 2-aPu residue, which is a part of a DNA substrate, during interaction with (**a**) WT Polβ [24] or polymorphic variants (**b**) L19P or (**c**) G66R at various dATP concentrations. [Enzyme] = 1.0 µM, [Gap_TÃ] = 0.5 µM. The dependence of the calculated values of the observed reaction rate constants on the dATP concentration is shown in panels (**d**) [24], (**e**), and (**f**) for the WT Polβ and polymorphic variants L19P and G66R, respectively.

Therefore, we registered the DNA conformational dynamics using a 2-aPu label in the course of the interaction of WT Polβ or its variants L19P and G66R with substrate Gap_TÃ DNA and dATP (Figure 12a–c).

Via this approach, two phases in fluorescence intensity changes in the 2-aPu residue were observed for both tested enzyme variants. The initial stage of rising fluorescence intensity of the 2-aPu residue ended approximately within 0.4–0.5 s in the case of polymorphic variants L19P and G66R but was faster (down to 0.1–0.3 s) for WT Polβ. Moreover, in the case of WT Polβ, the amplitude of 2-aPu fluorescence intensity growth was much higher, indicating that ternary-complex formation proceeds much more effectively with the WT enzyme. This difference reflects a substantial impact of the tested amino acid substitutions in the dRP-lyase domain on the formation of the ternary closed complex.

The accumulation of the reaction product caused the next change in fluorescence intensity of the 2-aPu residue (signal drop). It was found that for the G66R polymorphic variant, the signal reached a plateau more slowly than for the L19P polymorphic variant, indicating deceleration of the rate of the catalytic stage.

To determine observed dissociation constant *K*_d_, _app (dATP)_ of the enzyme–DNA complex toward dATP and polymerization constant *k*_pol_ (reflecting the rate of the chemical stage), the parts of the curves corresponding to the decrease in fluorescence intensity of the 2-aPu residue were fitted to Equation (4). The dependence of the observed rate constant on dATP concentration in the reaction was hyperbolic, and *k*_pol_ and *K*_d, app (dATP)_ were calculated via Equation (5) (Table 5).

Analysis of the obtained parameters (Table 5) revealed that polymerization constant *k*_pol_ for both polymorphic variants was ~2- and ~9-fold lower when compared with the WT enzyme. Additionally, dissociation constant *K*_d, app (dATP)_ for both polymorphic variants was 4–6-fold greater than this constant for the WT enzyme. These data implied that dNTP binding is considerably distorted by both substitutions, and moreover, the formation of the catalytic state is not efficient, resulting in a decrease in the chemical rate constant, especially for the G66R variant. These findings allow concluding that both Pro-19 and Arg-66 amino acid residues have an important role in the course of ternary closed complex formation and a substitution of these residues destabilizes the catalytic complex and diminishes the rate of the catalytic reaction.

**Figure 13:**
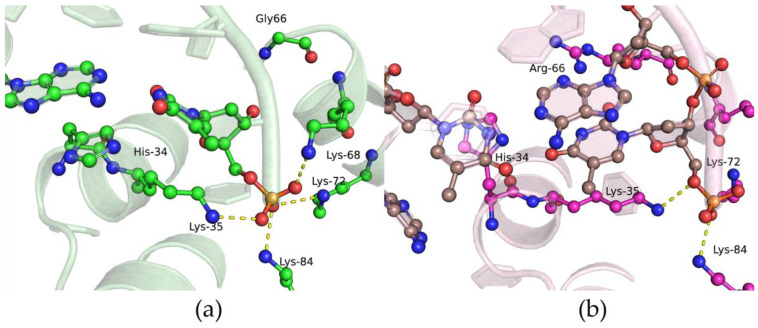
Snapshots of the dRP-lyase domain region of ternary protein–DNA–dNTP complex for the WT enzyme (**a**) and G66R variant (**b**). Salt bridges between amino acid sidechains and the sugar–phosphate backbone are presented as dashed lines.

Indeed, in the MD simulations (Figure 13) of the closed ternary complex formed by the G66R variant, it was noted that the bulky sidechain of residue Arg-66 causes displacement of the DNA primer, with the first downstream base pair being severed and the template base being everted and replaced by the His-34 sidechain. Therefore, it could be theorized that in the active site, these conformational changes induced by substitution G66R cause an appreciable reduction in nucleotidyltransferase activity of this enzyme.

## 3. Materials and Methods

### 3.1. Enzymes

**Figure 14:**
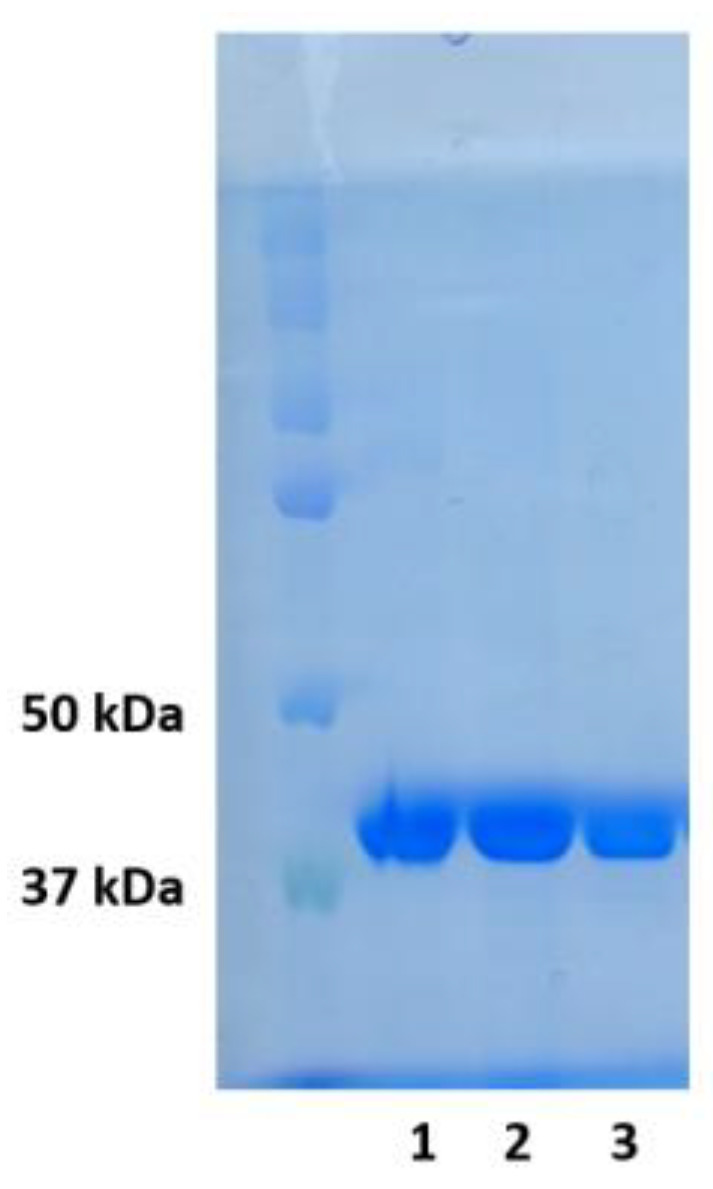
SDS-PAGE of purified fractions of WT Polβ and L19P and G66R variants. Line 1—WT Polβ, line 2—L19P, line 3—G66R.

Expression and purification of the protein was carried out using *Escherichia coli* Rosetta 2 cells transformed with the pET28-c plasmid carrying the full-length WT *POLB* gene or this gene with the corresponding SNP; for more details, refer to [41]. The purified enzymes (Figure 14) were stored in a buffer containing 50% glycerol at −20 °C.

### 3.2. DNA Substrates

**Figure 15:**
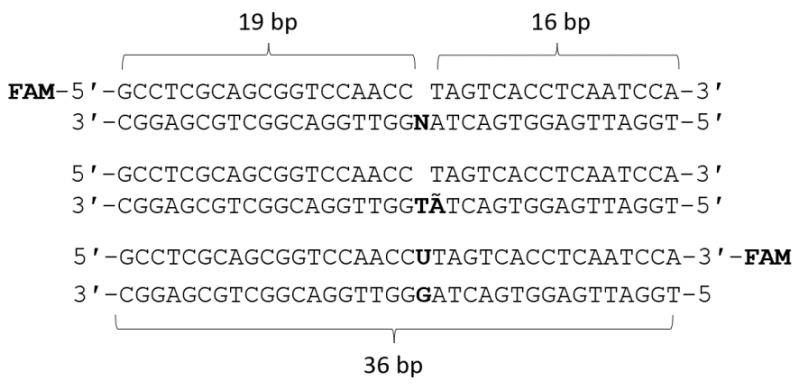
Sequences of 2′-oligodeoxyribonucleotides used in this work, where N = A/T/G/C, Ã = 2-aPu residue, and FAM = 6-fluorescein residue.

Two types of DNA substrates containing a Polβ-specific DNA lesion—a single-nucleotide gap—were used. Annealing was performed at 93 °C, and the mixture was cooled to room temperature. The resulting substrate was stored at −20 °C. The sequence of 2′-oligodeoxyribonucleotides containing a gap is shown in Figure 15.

### 3.3. Circular Dichroism (CD) Spectroscopy

The spectra were captured on a Jasco J-600 spectropolarimeter (Jasco, Tokyo, Japan). Full-length enzymes were present in the cuvette at a concentration of 1.0 M. In quartz cuvettes with a light path length of 0.1 mm, experiments were conducted in a buffer (50 mM Tris-HCl pH 7.5, 50 mM KCl, 1.0 mM EDTA, and 5.0 mM MgCl_2_). At room temperature, spectra with a bandwidth of 1.0 nm and a wavelength ranging from 190 to 260 nm were acquired. Automatic averaging was performed when the measurements were taken. An online tool for choosing and modeling protein CD spectra was used to describe the spectra [61].

### 3.4. Analysis of the Melting Point of the Enzymes

Melting points were measured by means of a Quant Studio 5 real-time PCR system (Applied Biosystems, Waltham, MA, USA) in PCR tubes using a thermal shift assay. Each tube contained 20 µL of a solution consisting of 50 µM protein, 50 mM Tris-HCl pH 7.5, 50 mM KCl, 1.0 mM EDTA, and 5.0 mM MgCl_2_, and 5X ProteOrange dye (Lumiprobe, Moscow, Russia). The temperature was constantly raised in steps of 0.028 °C from 25.1 to 99.9 °C. Fluorescence intensity of the ProteOrange dye was recorded using excitation at 470 nm and emission at 558 nm. Each melting point value was calculated using the Boltzmann sigmoid curve equation:

F = Fu + (Fb − Fu)/{1 + exp(T_m_ − x/slope)}, (1)

where F is ProteOrange fluorescence emission, x is temperature, Fu is baseline fluorescence at low temperature, Fb is maximum fluorescence at high temperature, the slope describes the steepness of the curve, and T_m_ is the melting point of the protein.

### 3.5. DNA-Binding Analysis

To determine the effect of the studied amino acid substitutions on the stage of enzyme binding to a DNA substrate containing a gap, the electrophoretic mobility shift assay was used. The reaction was carried out in a buffer composed of 50 mM Tris-HCl pH 7.5, 50 mM KCl, 1 mM Na_2_EDTA, 5 mM MgCl_2_, 1 mM DTT, and 7% glycerol. The recombinant enzymes were serially diluted; for the Polβ L19P polymorphic variant, the reaction was carried out in the concentration range from 57 nM to 7.3 μM, and for the G66R polymorphic variant, from 55.5 nM to 7.1 μM. The samples were incubated for 15 min at room temperature and applied to a nondenaturing 10% polyacrylamide gel (PAAG; the ratio of acrylamide to *N*,*N*′-methylenebisacrylamide was 75:1).

To determine the dissociation constant, the resultant gel was visualized in a VersaDoc gel-documenting system (Bio-Rad Laboratories, Hercules, CA, USA). The results were processed using Gel-Pro Analyzer 4 software (Media Cybernetics, Rockville, MD, USA). Dissociation constant *K*_d_ for each enzyme–DNA complex was computed in OriginPro 8 software via the following equation:

Formed complex (%) = Fu + (Fb − Fu)/{1 + (*K*_d_/[Polβ])h}, (2)

where h is the Hill coefficient, Fu is the correction for background illumination, and Fb is the maximum intensity of the complex.

### 3.6. Analysis of Polβ dRP-Lyase Activity

The dRP-lyase reaction substrate was generated using 3′-FAM-labeled 36 bp U-containing DNA substrate (5′-GCCTCGCAGCGGTCCAACC**U**TAGTCACCTCAATCCA-FAM/5′-TGGATTGAGGTGACTAGGGTTGGACGGCTGCGAGGC) treated with Udg and APE1 enzymes for 30 min at 37 °C in buffer containing 50 mM Tris-HCl pH 7.5, 50 mM KCl, 1 mM Na_2_EDTA, 5 mM Mg^2+^, 1 mM DTT, and 7% glycerol. The reaction mixture was purified by gel filtration with Sephadex G-25 400 μL columns. The resulting dRP-substrate was mixed with WT Polβ, L19P, or G66R variants, supplemented with 50 mM Tris-HCl pH 7.5, 50 mM KCl, 1 mM Na_2_EDTA, 5 mM Mg^2+^, 1 mM DTT, and 7% glycerol buffer, and incubated at 20 °C. The reaction was stopped by incubation on ice with 340 mM NaBH_4_ for 30 min. The excess of NaBH_4_ was deleted by gel filtration with Sephadex G-25 400 μL columns containing 7.5 M urea, 0.1% bromophenol blue, and 0.1% xylene cyanol dyes. The reaction product was separated in 20% TBE-urea PAAG at 500 V (constant V) for 5 h. The resulting gel was visualized in the VersaDoc gel-documenting system (Bio-Rad Laboratories, Hercules, CA, USA).

### 3.7. Analysis of Polβ Polymerase Activity

To determine the activity of the polymorphic variants of Polβ in the transferase reaction of the enzyme, separation of the products of the enzymatic reaction in a PAAG was used. The reaction was carried out via mixing of a DNA substrate solution, complementary dNTP, and an enzyme solution. In the final reaction mixture, the concentrations of the enzyme and DNA substrate were 0.5 µM, and dNTP was 5 µM. The reaction was carried out in a buffer consisting of 50 mM Tris-HCl pH 7.5, 50 mM KCl, 1 mM Na_2_EDTA, 5 mM MgCl_2_, 1 mM DTT, and 7% glycerol at 37 °C. From the reaction mixture, at certain time intervals, 5 μL of the solution was taken. The reaction was stopped by mixing with 5 μL of a stop solution (7.5 M urea, 25 mM EDTA, 0.1% xylene cyanol, and 0.1% bromophenol blue). The prepared samples were applied to a denaturing 15% PAAG. The resulting gel was visualized in the VersaDoc gel-documenting system (Bio-Rad Laboratories, Hercules, CA, USA). The data were processed using Gel-Pro Analyzer 4 software (Media Cybernetics, Rockville, MD, USA), and the degree of substrate conversion was determined by means of the ratio of peak areas of the product to the sum of the peak areas of the product and the peak of the initial substrate. A relevant characteristic of the polymerase activity of Polβ is the observed rate constant of the reaction of incorporation of various dNTPs into a synthesized DNA strand, *k*_obs_. The final calculation of the observed reaction rate constant was carried out in OriginPro 8 software via plotting of the dependence of the product accumulation on reaction time. The obtained data were fitted to the following equation:

[Product] = [S] × {1 − exp(−*k*_obs_ × t)}, (3)

where [S] is the initial concentration of the substrate, t is reaction time, and *k*_obs_ is the observed rate constant of the chemical reaction.

### 3.8. Registration of Conformational Changes in the DNA Substrate by the Stopped-Flow Method

Conformational changes in the DNA substrate containing a gap were recorded at a fluorescence excitation wavelength of 310 nm. Registration of conformational changes in the substrate DNA was carried out on an SX.20MV stopped-flow spectrophotometer (Applied Photophysics, Leatherhead, UK). To determine the influence of substitutions of amino acid residues on the catalytic step and the step of enzyme binding to dATP (formation of a ternary complex), the experiment was conducted by varying the concentration of dATP. The concentrations of the enzyme and DNA substrate were 1.0 and 0.5 μM, respectively; the reaction was carried out at 37 °C.

To determine polymerization reaction rate constant *k*_pol_ and observed constant *K*_d, app (dATP)_ of the dissociation of 2′-deoxyriboadenosine triphosphate from the enzyme–DNA complex, the region of the fluorescence curves corresponding to the slow stage of the decrease in fluorescence intensity of 2-aPu was fitted to the following equation [57]:

F = F_0_ + F_1_ × exp(−*k*_obs_ × t), (4)

where F is the observed 2-aPu fluorescence intensity signal, F_0_ is background fluorescence, F_1_ is a fluorescence parameter, and *k*_obs_ is the observed rate constant. From the obtained values of the observed rate constants, a dependence on the concentration of dATP was constructed. The resulting dependence was fitted to Equation (4), which made it possible to obtain parameters *k*_pol_ and *K*_d, app (dATP)_:

*k*_obs_ = *k*_pol_[dATP]/(*K*_d, app (dATP)_ + [dATP]), (5)

where *k*_obs_ is the observed rate constant of the reaction, *k*_pol_ is the rate constant of the polymerization reaction, and *K*_d, app (dATP)_ is the dissociation constant of the enzyme–DNA–dATP complex.

### 3.9. Molecular Dynamics (MD) Simulations

The human Polβ apoenzyme structure was modeled by means of the crystal structure of rat Polβ [62]. An N-terminal dRP-lyase domain fragment was derived from the NMR structure [63]. Models of the open binary Polβ–DNA complex and ternary closed Polβ–DNA–dNTP complex were based on crystal structures of human Polβ complexes [17,64], with DNA edited to match experimental sequences. Homology and unstructured region modeling were performed using Modeller [65]. Protein protonation states were assigned by the PDB2PQR server with PROPKA [66,67]. Simulations were run with the help of the GROMACS MD package. A simulation box was set up with TIP3P water and 50 mM KCl JC ions [68,69]. Octahedral dummy model treatment was chosen for active-site magnesium ions [70]. The protein and the DNA primer were parametrized with the AMBER 14SB-OL15 force field set [71,72,73]. RESP charges for nucleoside triphosphates were assigned using the R.E.D. Server via an established approach [74,75]. Force field parameters were converted with ACPYPE [76]. The cutoff of nonbonded interactions was set to 0.8 nm, and long-range electrostatic interactions were treated via the PME method [77]. Bonds of hydrogen atoms were constrained using LINCS [78]. Flat-bottom potential restraints were applied to hydrogen-bonded heavy atoms of terminal base pairs in the truncated DNA primers. Steepest descent energy minimization was followed by 1 ns NVT and NPT equilibrations with heavy atom restraints. Unrestrained MD simulations were run in triplicate for 0.5 and 2 μs for the N-terminal fragment, using a V-rescale thermostat and C-rescale barostat [79,80]. Resultant trajectories were processed with the integrated GROMACS toolset. PCA calculations were performed and plotted in NMWiz.

## 4. Conclusions

DNA polymerase β is engaged in numerous cellular processes that are essential for the cell to function normally. Filling in DNA gaps left by other enzymes, including DNA repair enzymes or exogenous and endogenous factors, is one of Polβ’s key roles. Due to the possible impact of SNP-induced amino acid substitutions within the enzyme globule on stages of the enzyme’s mechanism of action, the presence of SNPs in the *POLΒ* gene itself may result in a reduction in the efficiency of the whole DNA repair process. Here, we tested the DNA- and dNTP-binding abilities as well as catalytic activity of two SNP variants of human Polβ, which contain amino acid substitutions L19P or G66R. By a thermal shift assay and the CD method, it was found that substitutions L19P and G66R have a negligible effect on the thermal stability and secondary structure of Polβ. EMSA revealed that these SNP-induced substitutions diminish the efficiency of formation of the enzyme complex with DNA containing a 1 nt gap by 3- and 4-fold. A kinetic analysis of the incorporation of the incoming nucleotide into model DNA substrates indicated that substitutions L19P and G66R reduce the transferase activity of Polβ by decreasing observed rate constant *k*_obs_ at least 30- and 5-fold, respectively. Changes in fluorescence intensity of a 2-aPu residue located in a DNA substrate, after interaction with the tested enzyme variants at different dATP concentrations, made it possible to find that the Polβ L19P and G66R polymorphic variants have lower efficiency of formation of the Polβ–DNA–dATP ternary complex. In addition, the L19P variant had reduced dRP-lyase activity, while the G66R variant had activity comparable to the WT Polβ. Another known polymorphic variant of Polβ, L22P, had a similar effect on the enzymatic activity of an enzyme [35]. It was found that L19P, like the L22P variant, has lower DNA-binding affinity. In the case of the L19P variant, a similar effect on the change in the position of the α1-helix can be assumed. Along with the L22P variant, a decrease in dRP-lyase activity was also observed for the L19P variant. In primer extension experiments, it was shown that the L19P substitution leads to reduced polymerase activity; similar data were obtained for the L22P variant [35]. It can be assumed that, by analogy with the L22P variant, the L19P variant has a reduced ability to support BER. Overall, our data show the influence of the tested SNPs on each stage of the enzymatic interaction of Polβ with DNA, thus indicating the high potential of these SNPs to act as a source of genetic instability and increase the risk of mutations in the genome.

## Figures and Tables

**Table 1:** Occurrence of Polβ mutations in the dRP-lyase domain for various types of tumors.

Amino Acid Substitution	COSMIC (https://cancer.sanger.ac.uk, accessed on 21 February 2021)	cBioportal (https://www.cbioportal.org, accessed on 21 February 2021)	Hivebiochemistry (https://hive.biochemistry.gwu.edu, accessed on 21 February 2021)
M1V			Liver cancer
S2I			Blastoma
S2R			Liver cancer
R4L	Choriocarcinoma		
A6S	Small cell lung carcinoma		
Q8R	Non–small cell lung carcinoma		
E9D		Uterine endometrioid carcinoma	Uterine cancer
L11F	Glioblastoma		
L11V	Colon adenocarcinoma		Uterine cancer
G13V		Astrocytoma	Melanoma
D17N	Thyroid carcinoma	Papillary thyroid cancer	Malignant glioma
M18I	Colon adenocarcinoma, rectal adenocarcinoma	Rectal adenocarcinoma	Colorectal cancer, thyroid carcinoma
L22F	Bile duct adenocarcinoma		
A23T			Melanoma
A23V	Squamous cell lung carcinoma	Lung squamous cell carcinoma	Kidney cancer
K27R	Lung adenocarcinoma	Lung adenocarcinoma	Lung cancer
N37H	Liver carcinoma		Blastoma, lung cancer
A38S			Liver cancer
S44C	Stomach adenocarcinoma	Tubular stomach adenocarcinoma	Liver cancer
S44F			Stomach cancer
Y49S	Prostate adenocarcinoma		Colorectal cancer
P50L	Prostate carcinoma		Prostate cancer
A59S			Prostate cancer
L62F	Thyroid carcinoma		Liver cancer
E71K	Endometrioid carcinoma	Uterine serous carcinoma	Thyroid carcinoma
F76C	Endometrioid carcinoma	Uterine endometrioid carcinoma	Uterine cancer
G80R	Serous carcinoma		Uterine cancer
R83C	Endometrioid carcinoma, lung adenocarcinoma, stomach adenocarcinoma	Uterine endometrioid carcinoma, lung adenocarcinoma, tubular stomach adenocarcinoma	Germ cell cancer, lung cancer, gastric cancer
R83H	Cecum adenocarcinoma		
L85V	Esophageal squamous cell carcinoma		
E86G	Colon adenocarcinoma		Uterine cancer

**Table 3:** Calculated dissociation constants *K*_d_ (μM) of the enzyme–DNA complexes.

Substrate	WT Polβ [24]	Polβ L19P	Polβ G66R
Gap_A	0.38 ± 0.02	0.86 ± 0.04	1.7 ± 0.2
Gap_T	0.33 ± 0.03	2.0 ± 0.4	0.9 ± 0.1
Gap_G	0.59 ± 0.07	1.3 ± 0.1	2.0 ± 0.3
Gap_C	0.38 ± 0.03	1.1 ± 0.1	1.0 ± 0.1

**Table 4:** Observed reaction rate constants *k*_obs_ for the incorporation of a complimentary nucleotide into the tested DNA substrates containing a 1 nt gap.

*k*_obs_, s^−1^	WT Polβ [24]	Polβ L19P	Polβ G66R
Gap_A	0.33 ± 0.03	0.005 ± 0.001	0.052 ± 0.004
Gap_T	0.32 ± 0.04	0.009 ± 0.001	0.048 ± 0.006
Gap_G	0.25 ± 0.02	0.008 ± 0.001	0.047 ± 0.002
Gap_C	0.34 ± 0.02	0.011 ± 0.001	0.065 ± 0.008

**Table 5:** Chemical reaction rate constants *k*_pol_ and observed dissociation constants *K*_d_, _app (dATP)_.

Enzyme	WT Polβ [41]	Polβ L19P	Polβ G66R
*k*_pol_, s^−1^	0.93 ± 0.05	0.54 ± 0.22	0.10 ± 0.01
*K*_d_, _app(dATP)_, μM	16 ± 3	101 ± 36	74 ± 19

## Data Availability

Raw experimental data are available from O.A.K. and A.A.K. upon request. Tel.: +7-(383)-363-5174, e-mail: kladova@niboch.nsc.ru (O.A.K.); sandra-k@niboch.nsc.ru (A.A.K.).
